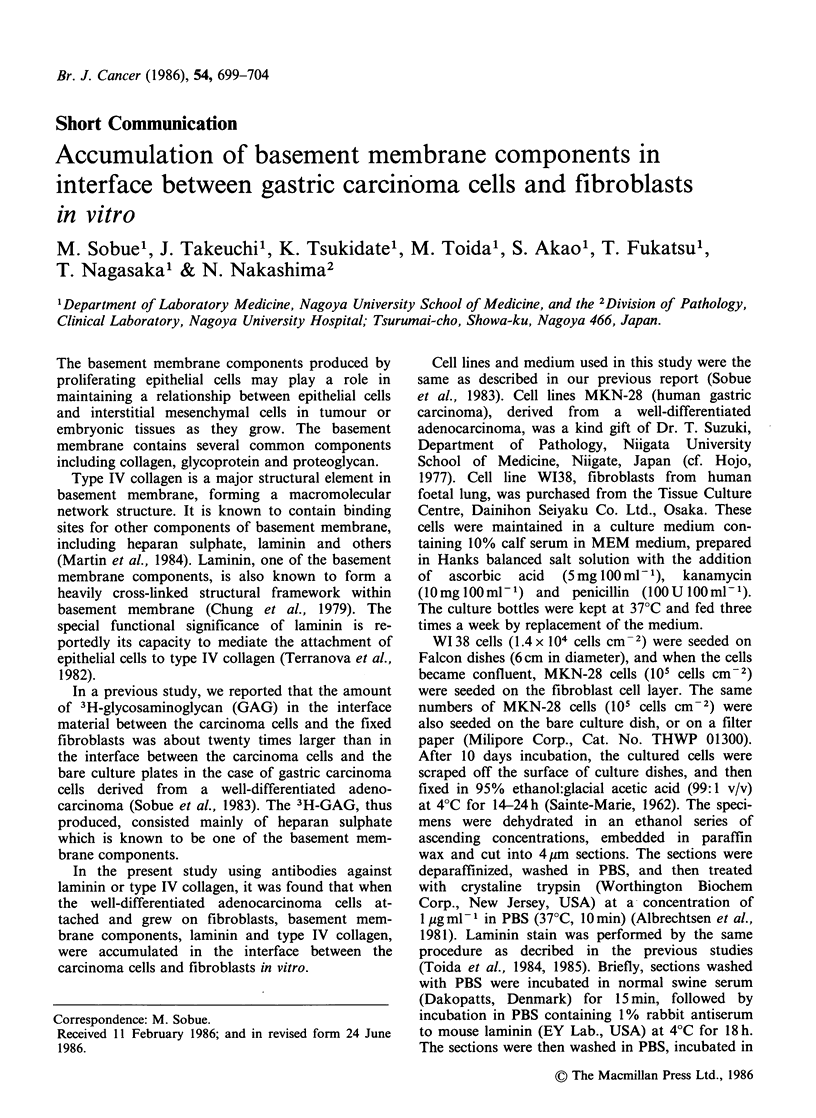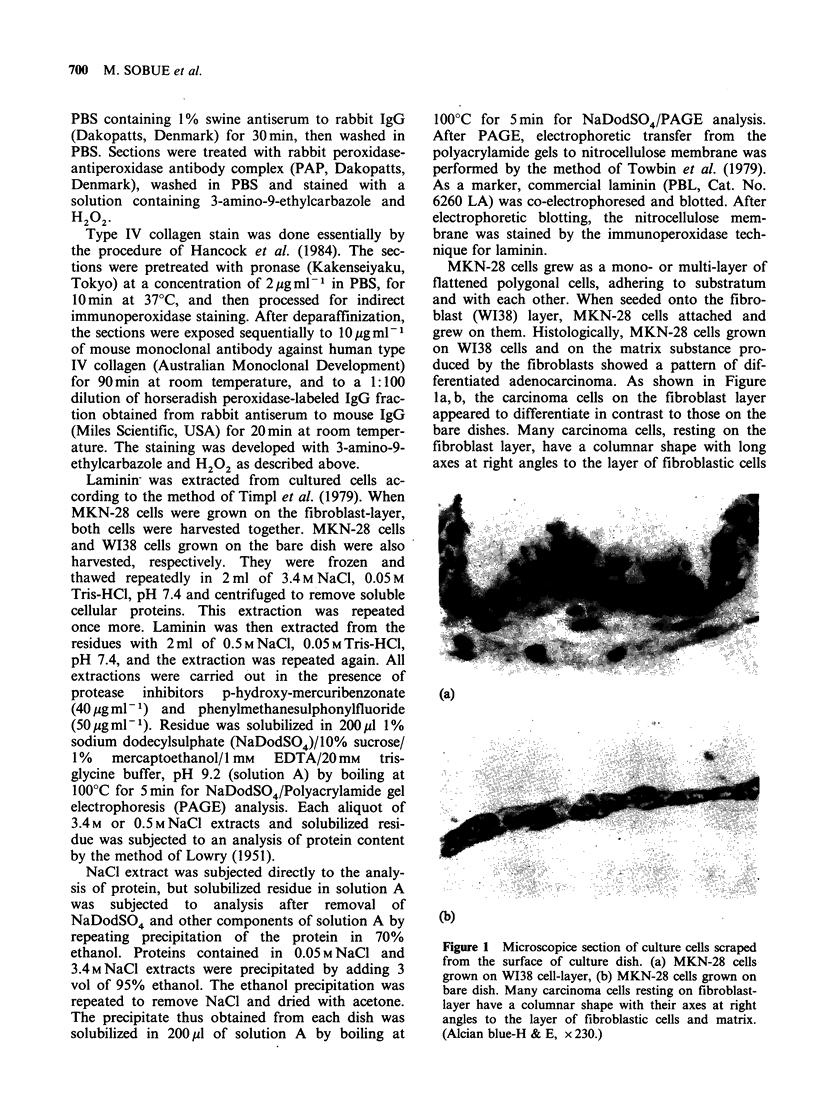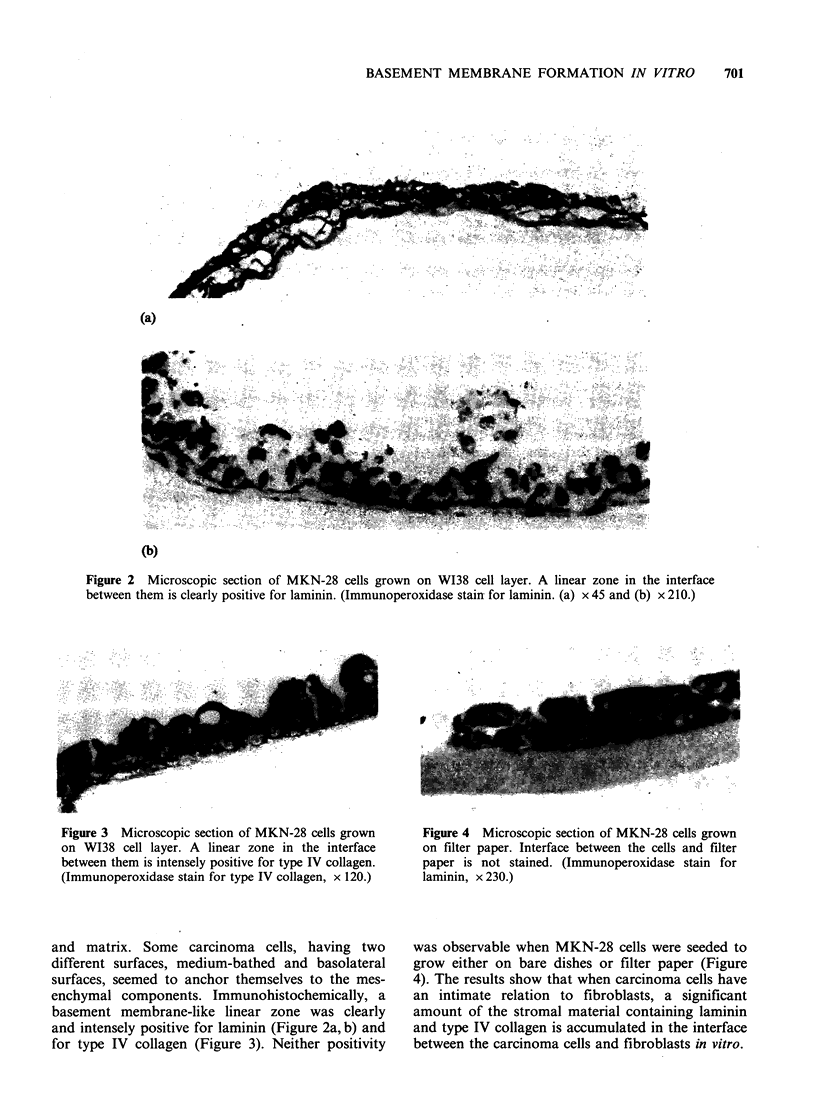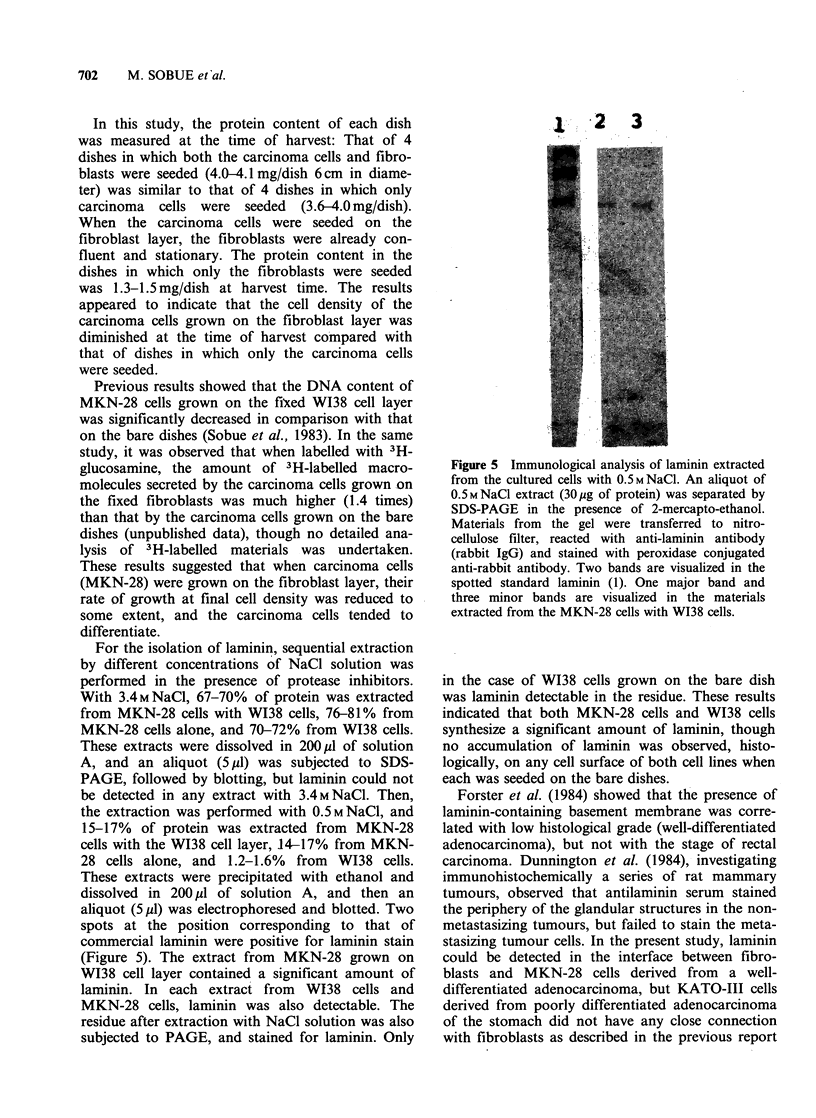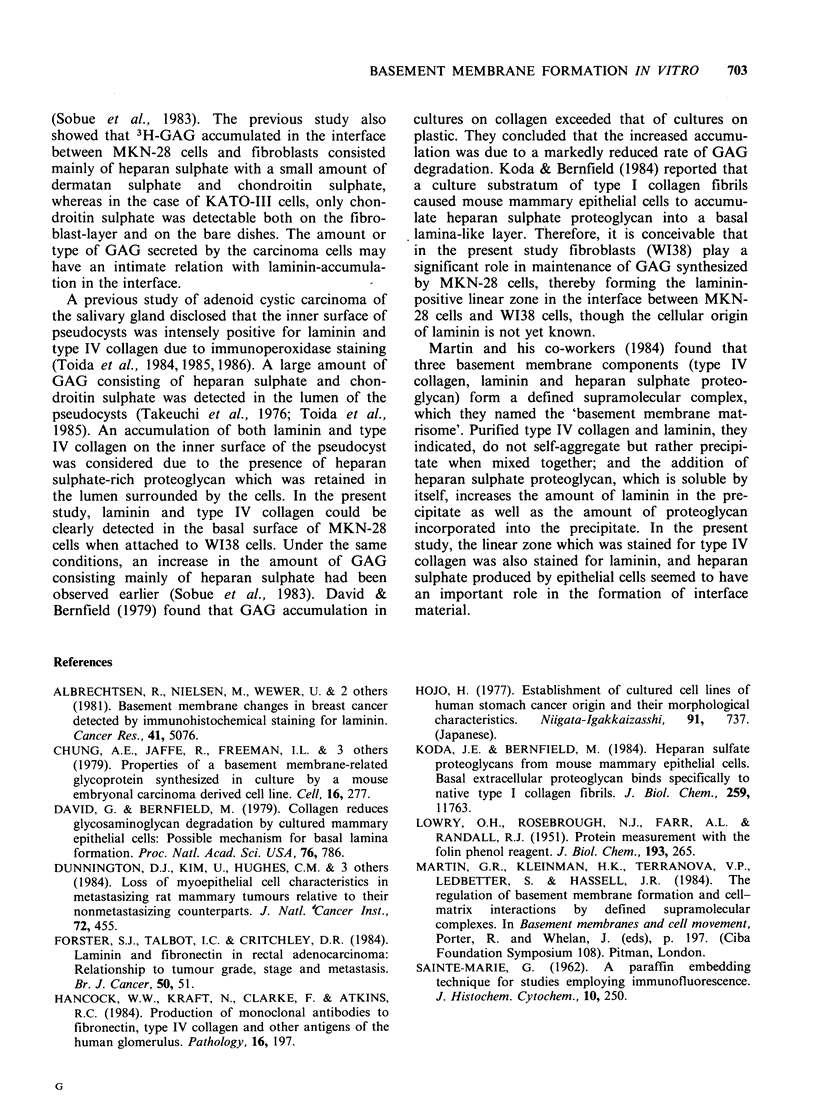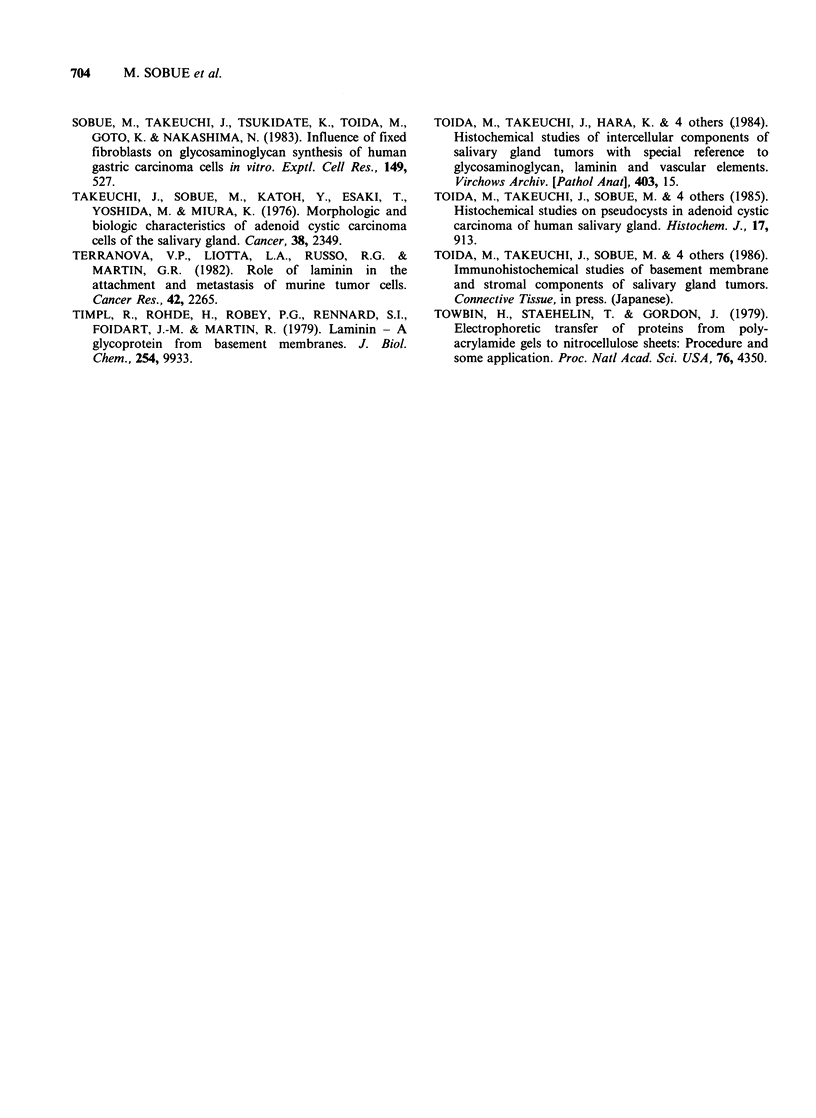# Accumulation of basement membrane components in interface between gastric carcinoma cells and fibroblasts in vitro.

**DOI:** 10.1038/bjc.1986.229

**Published:** 1986-10

**Authors:** M. Sobue, J. Takeuchi, K. Tsukidate, M. Toida, S. Akao, T. Fukatsu, T. Nagasaka, N. Nakashima

## Abstract

**Images:**


*Br. J. Cancer* (1986), **54**, 699–704

**Short Communication**

### Accumulation of basement membrane components in interface between gastric carcinoma cells and fibroblasts *in vitro*

M. Sobue[1], J. Takeuchi[1], K. Tsukidate[1], M. Toida[1], S. Akao[1], T. Fukatsu[1], T. Nagasaka[1] & N. Nakashima[2]

[1]*Department of Laboratory Medicine, Nagoya University School of Medicine, and the* [2]*Division of Pathology, Clinical Laboratory, Nagoya University Hospital; Tsurumai-cho, Showa-ku, Nagoya 466, Japan.*

The basement membrane components produced by proliferating epithelial cells may play a role in maintaining a relationship between epithelial cells and interstitial mesenchymal cells in tumour or embryonic tissues as they grow. The basement membrane contains several common components including collagen, glycoprotein and proteoglycan.

Type IV collagen is a major structural element in basement membrane, forming a macromolecular network structure. It is known to contain binding sites for other components of basement membrane, including heparan sulphate, laminin and others (Martin *et al.*, 1984). Laminin, one of the basement membrane components, is also known to form a heavily cross-linked structural framework within basement membrane (Chung *et al.*, 1979). The special functional significance of laminin is reportedly its capacity to mediate the attachment of epithelial cells to type IV collagen (Terranova *et al.*, 1982).

In a previous study, we reported that the amount of $^3$H-glycosaminoglycan (GAG) in the interface material between the carcinoma cells and the fixed fibroblasts was about twenty times larger than in the interface between the carcinoma cells and the bare culture plates in the case of gastric carcinoma cells derived from a well-differentiated adenocarcinoma (Sobue *et al.*, 1983). The $^3$H-GAG, thus produced, consisted mainly of heparan sulphate which is known to be one of the basement membrane components.

In the present study using antibodies against laminin or type IV collagen, it was found that when the well-differentiated adenocarcinoma cells attached and grew on fibroblasts, basement membrane components, laminin and type IV collagen, were accumulated in the interface between the carcinoma cells and fibroblasts *in vitro*.

Correspondence: M. Sobue.
Received 11 February 1986; and in revised form 24 June 1986.

Cell lines and medium used in this study were the same as described in our previous report (Sobue *et al.*, 1983). Cell lines MKN-28 (human gastric carcinoma), derived from a well-differentiated adenocarcinoma, was a kind gift of Dr. T. Suzuki, Department of Pathology, Niigata University School of Medicine, Niigate, Japan (cf. Hojo, 1977). Cell line WI38, fibroblasts from human foetal lung, was purchased from the Tissue Culture Centre, Dainihon Seiyaku Co. Ltd., Osaka. These cells were maintained in a culture medium containing 10% calf serum in MEM medium, prepared in Hanks balanced salt solution with the addition of ascorbic acid ($5\,mg\,100\,ml^{-1}$), kanamycin ($10\,mg\,100\,ml^{-1}$) and penicillin ($100\,U\,100\,ml^{-1}$). The culture bottles were kept at 37°C and fed three times a week by replacement of the medium.

WI 38 cells ($1.4\times10^4$ cells $cm^{-2}$) were seeded on Falcon dishes (6 cm in diameter), and when the cells became confluent, MKN-28 cells ($10^5$ cells $cm^{-2}$) were seeded on the fibroblast cell layer. The same numbers of MKN-28 cells ($10^5$ cells $cm^{-2}$) were also seeded on the bare culture dish, or on a filter paper (Milipore Corp., Cat. No. THWP 01300). After 10 days incubation, the cultured cells were scraped off the surface of culture dishes, and then fixed in 95% ethanol:glacial acetic acid (99:1 v/v) at 4°C for 14–24 h (Sainte-Marie, 1962). The specimens were dehydrated in an ethanol series of ascending concentrations, embedded in paraffin wax and cut into 4 $\mu$m sections. The sections were deparaffinized, washed in PBS, and then treated with crystaline trypsin (Worthington Biochem Corp., New Jersey, USA) at a concentration of $1\,\mu g\,ml^{-1}$ in PBS (37°C, 10 min) (Albrechtsen *et al.*, 1981). Laminin stain was performed by the same procedure as decribed in the previous studies (Toida *et al.*, 1984, 1985). Briefly, sections washed with PBS were incubated in normal swine serum (Dakopatts, Denmark) for 15 min, followed by incubation in PBS containing 1% rabbit antiserum to mouse laminin (EY Lab., USA) at 4°C for 18 h. The sections were then washed in PBS, incubated in

© The Macmillan Press Ltd., 1986

PBS containing 1% swine antiserum to rabbit IgG (Dakopatts, Denmark) for 30 min, then washed in PBS. Sections were treated with rabbit peroxidase-antiperoxidase antibody complex (PAP, Dakopatts, Denmark), washed in PBS and stained with a solution containing 3-amino-9-ethylcarbazole and $H_2O_2$.

Type IV collagen stain was done essentially by the procedure of Hancock *et al.* (1984). The sections were pretreated with pronase (Kakenseiyaku, Tokyo) at a concentration of $2\,\mu g\,ml^{-1}$ in PBS, for 10 min at 37°C, and then processed for indirect immunoperoxidase staining. After deparaffinization, the sections were exposed sequentially to $10\,\mu g\,ml^{-1}$ of mouse monoclonal antibody against human type IV collagen (Australian Monoclonal Development) for 90 min at room temperature, and to a 1:100 dilution of horseradish peroxidase-labeled IgG fraction obtained from rabbit antiserum to mouse IgG (Miles Scientific, USA) for 20 min at room temperature. The staining was developed with 3-amino-9-ethylcarbazole and $H_2O_2$ as described above.

Laminin was extracted from cultured cells according to the method of Timpl *et al.* (1979). When MKN-28 cells were grown on the fibroblast-layer, both cells were harvested together. MKN-28 cells and WI38 cells grown on the bare dish were also harvested, respectively. They were frozen and thawed repeatedly in 2 ml of 3.4 M NaCl, 0.05 M Tris-HCl, pH 7.4 and centrifuged to remove soluble cellular proteins. This extraction was repeated once more. Laminin was then extracted from the residues with 2 ml of 0.5 M NaCl, 0.05 M Tris-HCl, pH 7.4, and the extraction was repeated again. All extractions were carried out in the presence of protease inhibitors p-hydroxy-mercuribenzonate ($40\,\mu g\,ml^{-1}$) and phenylmethanesulphonylfluoride ($50\,\mu g\,ml^{-1}$). Residue was solubilized in 200 μl 1% sodium dodecylsulphate ($NaDodSO_4$)/10% sucrose/ 1% mercaptoethanol/1 mM EDTA/20 mM tris-glycine buffer, pH 9.2 (solution A) by boiling at 100°C for 5 min for $NaDodSO_4$/Polyacrylamide gel electrophoresis (PAGE) analysis. Each aliquot of 3.4 M or 0.5 M NaCl extracts and solubilized residue was subjected to an analysis of protein content by the method of Lowry (1951).

NaCl extract was subjected directly to the analysis of protein, but solubilized residue in solution A was subjected to analysis after removal of $NaDodSO_4$ and other components of solution A by repeating precipitation of the protein in 70% ethanol. Proteins contained in 0.05 M NaCl and 3.4 M NaCl extracts were precipitated by adding 3 vol of 95% ethanol. The ethanol precipitation was repeated to remove NaCl and dried with acetone. The precipitate thus obtained from each dish was solubilized in 200 μl of solution A by boiling at 100°C for 5 min for $NaDodSO_4$/PAGE analysis. After PAGE, electrophoretic transfer from the polyacrylamide gels to nitrocellulose membrane was performed by the method of Towbin *et al.* (1979). As a marker, commercial laminin (PBL, Cat. No. 6260 LA) was co-electrophoresed and blotted. After electrophoretic blotting, the nitrocellulose membrane was stained by the immunoperoxidase technique for laminin.

MKN-28 cells grew as a mono- or multi-layer of flattened polygonal cells, adhering to substratum and with each other. When seeded onto the fibroblast (WI38) layer, MKN-28 cells attached and grew on them. Histologically, MKN-28 cells grown on WI38 cells and on the matrix substance produced by the fibroblasts showed a pattern of differentiated adenocarcinoma. As shown in Figure 1a, b, the carcinoma cells on the fibroblast layer appeared to differentiate in contrast to those on the bare dishes. Many carcinoma cells, resting on the fibroblast layer, have a columnar shape with long axes at right angles to the layer of fibroblastic cells

(a)

(b)

**Figure 1** Microscopice section of culture cells scraped from the surface of culture dish. (a) MKN-28 cells grown on WI38 cell-layer, (b) MKN-28 cells grown on bare dish. Many carcinoma cells resting on fibroblast-layer have a columnar shape with their axes at right angles to the layer of fibroblastic cells and matrix. (Alcian blue-H & E, ×230.)

**Figure 2** Microscopic section of MKN-28 cells grown on WI38 cell layer. A linear zone in the interface between them is clearly positive for laminin. (Immunoperoxidase stain for laminin. (a) ×45 and (b) ×210.)

**Figure 3** Microscopic section of MKN-28 cells grown on WI38 cell layer. A linear zone in the interface between them is intensely positive for type IV collagen. (Immunoperoxidase stain for type IV collagen, ×120.)

**Figure 4** Microscopic section of MKN-28 cells grown on filter paper. Interface between the cells and filter paper is not stained. (Immunoperoxidase stain for laminin, ×230.)

and matrix. Some carcinoma cells, having two different surfaces, medium-bathed and basolateral surfaces, seemed to anchor themselves to the mesenchymal components. Immunohistochemically, a basement membrane-like linear zone was clearly and intensely positive for laminin (Figure 2a, b) and for type IV collagen (Figure 3). Neither positivity was observable when MKN-28 cells were seeded to grow either on bare dishes or filter paper (Figure 4). The results show that when carcinoma cells have an intimate relation to fibroblasts, a significant amount of the stromal material containing laminin and type IV collagen is accumulated in the interface between the carcinoma cells and fibroblasts *in vitro*.

In this study, the protein content of each dish was measured at the time of harvest: That of 4 dishes in which both the carcinoma cells and fibroblasts were seeded (4.0–4.1 mg/dish 6 cm in diameter) was similar to that of 4 dishes in which only carcinoma cells were seeded (3.6–4.0 mg/dish). When the carcinoma cells were seeded on the fibroblast layer, the fibroblasts were already confluent and stationary. The protein content in the dishes in which only the fibroblasts were seeded was 1.3–1.5 mg/dish at harvest time. The results appeared to indicate that the cell density of the carcinoma cells grown on the fibroblast layer was diminished at the time of harvest compared with that of dishes in which only the carcinoma cells were seeded.

Previous results showed that the DNA content of MKN-28 cells grown on the fixed WI38 cell layer was significantly decreased in comparison with that on the bare dishes (Sobue et al., 1983). In the same study, it was observed that when labelled with $^3$H-glucosamine, the amount of $^3$H-labelled macromolecules secreted by the carcinoma cells grown on the fixed fibroblasts was much higher (1.4 times) than that by the carcinoma cells grown on the bare dishes (unpublished data), though no detailed analysis of $^3$H-labelled materials was undertaken. These results suggested that when carcinoma cells (MKN-28) were grown on the fibroblast layer, their rate of growth at final cell density was reduced to some extent, and the carcinoma cells tended to differentiate.

For the isolation of laminin, sequential extraction by different concentrations of NaCl solution was performed in the presence of protease inhibitors. With 3.4 M NaCl, 67–70% of protein was extracted from MKN-28 cells with WI38 cells, 76–81% from MKN-28 cells alone, and 70–72% from WI38 cells. These extracts were dissolved in 200 $\mu$l of solution A, and an aliquot (5 $\mu$l) was subjected to SDS-PAGE, followed by blotting, but laminin could not be detected in any extract with 3.4 M NaCl. Then, the extraction was performed with 0.5 M NaCl, and 15–17% of protein was extracted from MKN-28 cells with the WI38 cell layer, 14–17% from MKN-28 cells alone, and 1.2–1.6% from WI38 cells. These extracts were precipitated with ethanol and dissolved in 200 $\mu$l of solution A, and then an aliquot (5 $\mu$l) was electrophoresed and blotted. Two spots at the position corresponding to that of commercial laminin were positive for laminin stain (Figure 5). The extract from MKN-28 grown on WI38 cell layer contained a significant amount of laminin. In each extract from WI38 cells and MKN-28 cells, laminin was also detectable. The residue after extraction with NaCl solution was also subjected to PAGE, and stained for laminin. Only

**Figure 5** Immunological analysis of laminin extracted from the cultured cells with 0.5 M NaCl. An aliquot of 0.5 M NaCl extract (30 $\mu$g of protein) was separated by SDS-PAGE in the presence of 2-mercapto-ethanol. Materials from the gel were transferred to nitrocellulose filter, reacted with anti-laminin antibody (rabbit IgG) and stained with peroxidase conjugated anti-rabbit antibody. Two bands are visualized in the spotted standard laminin (1). One major band and three minor bands are visualized in the materials extracted from the MKN-28 cells with WI38 cells.

in the case of WI38 cells grown on the bare dish was laminin detectable in the residue. These results indicated that both MKN-28 cells and WI38 cells synthesize a significant amount of laminin, though no accumulation of laminin was observed, histologically, on any cell surface of both cell lines when each was seeded on the bare dishes.

Forster et al. (1984) showed that the presence of laminin-containing basement membrane was correlated with low histological grade (well-differentiated adenocarcinoma), but not with the stage of rectal carcinoma. Dunnington et al. (1984), investigating immunohistochemically a series of rat mammary tumours, observed that antilaminin serum stained the periphery of the glandular structures in the non-metastasizing tumours, but failed to stain the metastasizing tumour cells. In the present study, laminin could be detected in the interface between fibroblasts and MKN-28 cells derived from a well-differentiated adenocarcinoma, but KATO-III cells derived from poorly differentiated adenocarcinoma of the stomach did not have any close connection with fibroblasts as described in the previous report

(Sobue *et al.*, 1983). The previous study also showed that $^3$H-GAG accumulated in the interface between MKN-28 cells and fibroblasts consisted mainly of heparan sulphate with a small amount of dermatan sulphate and chondroitin sulphate, whereas in the case of KATO-III cells, only chondroitin sulphate was detectable both on the fibroblast-layer and on the bare dishes. The amount or type of GAG secreted by the carcinoma cells may have an intimate relation with laminin-accumulation in the interface.

A previous study of adenoid cystic carcinoma of the salivary gland disclosed that the inner surface of pseudocysts was intensely positive for laminin and type IV collagen due to immunoperoxidase staining (Toida *et al.*, 1984, 1985, 1986). A large amount of GAG consisting of heparan sulphate and chondroitin sulphate was detected in the lumen of the pseudocysts (Takeuchi *et al.*, 1976; Toida *et al.*, 1985). An accumulation of both laminin and type IV collagen on the inner surface of the pseudocyst was considered due to the presence of heparan sulphate-rich proteoglycan which was retained in the lumen surrounded by the cells. In the present study, laminin and type IV collagen could be clearly detected in the basal surface of MKN-28 cells when attached to WI38 cells. Under the same conditions, an increase in the amount of GAG consisting mainly of heparan sulphate had been observed earlier (Sobue *et al.*, 1983). David & Bernfield (1979) found that GAG accumulation in cultures on collagen exceeded that of cultures on plastic. They concluded that the increased accumulation was due to a markedly reduced rate of GAG degradation. Koda & Bernfield (1984) reported that a culture substratum of type I collagen fibrils caused mouse mammary epithelial cells to accumulate heparan sulphate proteoglycan into a basal lamina-like layer. Therefore, it is conceivable that in the present study fibroblasts (WI38) play a significant role in maintenance of GAG synthesized by MKN-28 cells, thereby forming the laminin-positive linear zone in the interface between MKN-28 cells and WI38 cells, though the cellular origin of laminin is not yet known.

Martin and his co-workers (1984) found that three basement membrane components (type IV collagen, laminin and heparan sulphate proteoglycan) form a defined supramolecular complex, which they named the 'basement membrane matrisome'. Purified type IV collagen and laminin, they indicated, do not self-aggregate but rather precipitate when mixed together; and the addition of heparan sulphate proteoglycan, which is soluble by itself, increases the amount of laminin in the precipitate as well as the amount of proteoglycan incorporated into the precipitate. In the present study, the linear zone which was stained for type IV collagen was also stained for laminin, and heparan sulphate produced by epithelial cells seemed to have an important role in the formation of interface material.

### References

ALBRECHTSEN, R., NIELSEN, M., WEWER, U. & 2 others (1981). Basement membrane changes in breast cancer detected by immunohistochemical staining for laminin. *Cancer Res.*, **41,** 5076.

CHUNG, A.E., JAFFE, R., FREEMAN, I.L. & 3 others (1979). Properties of a basement membrane-related glycoprotein synthesized in culture by a mouse embryonal carcinoma derived cell line. *Cell*, **16,** 277.

DAVID, G. & BERNFIELD, M. (1979). Collagen reduces glycosaminoglycan degradation by cultured mammary epithelial cells: Possible mechanism for basal lamina formation. *Proc. Natl. Acad. Sci. USA*, **76,** 786.

DUNNINGTON, D.J., KIM, U., HUGHES, C.M. & 3 others (1984). Loss of myoepithelial cell characteristics in metastasizing rat mammary tumours relative to their nonmetastasizing counterparts. *J. Natl. Cancer Inst.*, **72,** 455.

FORSTER, S.J., TALBOT, I.C. & CRITCHLEY, D.R. (1984). Laminin and fibronectin in rectal adenocarcinoma: Relationship to tumour grade, stage and metastasis. *Br. J. Cancer*, **50,** 51.

HANCOCK, W.W., KRAFT, N., CLARKE, F. & ATKINS, R.C. (1984). Production of monoclonal antibodies to fibronectin, type IV collagen and other antigens of the human glomerulus. *Pathology*, **16,** 197.

HOJO, H. (1977). Establishment of cultured cell lines of human stomach cancer origin and their morphological characteristics. *Niigata-Igakkaizasshi*, **91,** 737. (Japanese).

KODA, J.E. & BERNFIELD, M. (1984). Heparan sulfate proteoglycans from mouse mammary epithelial cells. Basal extracellular proteoglycan binds specifically to native type I collagen fibrils. *J. Biol. Chem.*, **259,** 11763.

LOWRY, O.H., ROSEBROUGH, N.J., FARR, A.L. & RANDALL, R.J. (1951). Protein measurement with the folin phenol reagent. *J. Biol. Chem.*, **193,** 265.

MARTIN, G.R., KLEINMAN, H.K., TERRANOVA, V.P., LEDBETTER, S. & HASSELL, J.R. (1984). The regulation of basement membrane formation and cell–matrix interactions by defined supramolecular complexes. In *Basement membranes and cell movement*, Porter, R. and Whelan, J. (eds), p. 197. (Ciba Foundation Symposium 108). Pitman, London.

SAINTE-MARIE, G. (1962). A paraffin embedding technique for studies employing immunofluorescence. *J. Histochem. Cytochem.*, **10,** 250.

SOBUE, M., TAKEUCHI, J., TSUKIDATE, K., TOIDA, M., GOTO, K. & NAKASHIMA, N. (1983). Influence of fixed fibroblasts on glycosaminoglycan synthesis of human gastric carcinoma cells *in vitro*. *Exptl. Cell Res.*, **149,** 527.

TAKEUCHI, J., SOBUE, M., KATOH, Y., ESAKI, T., YOSHIDA, M. & MIURA, K. (1976). Morphologic and biologic characteristics of adenoid cystic carcinoma cells of the salivary gland. *Cancer*, **38,** 2349.

TERRANOVA, V.P., LIOTTA, L.A., RUSSO, R.G. & MARTIN, G.R. (1982). Role of laminin in the attachment and metastasis of murine tumor cells. *Cancer Res.*, **42,** 2265.

TIMPL, R., ROHDE, H., ROBEY, P.G., RENNARD, S.I., FOIDART, J.-M. & MARTIN, R. (1979). Laminin – A glycoprotein from basement membranes. *J. Biol. Chem.*, **254,** 9933.

TOIDA, M., TAKEUCHI, J., HARA, K. & 4 others (1984). Histochemical studies of intercellular components of salivary gland tumors with special reference to glycosaminoglycan, laminin and vascular elements. *Virchows Archiv.* [*Pathol Anat*], **403,** 15.

TOIDA, M., TAKEUCHI, J., SOBUE, M. & 4 others (1985). Histochemical studies on pseudocysts in adenoid cystic carcinoma of human salivary gland. *Histochem. J.*, **17,** 913.

TOIDA, M., TAKEUCHI, J., SOBUE, M. & 4 others (1986). Immunohistochemical studies of basement membrane and stromal components of salivary gland tumors. *Connective Tissue*, in press. (Japanese).

TOWBIN, H., STAEHELIN, T. & GORDON, J. (1979). Electrophoretic transfer of proteins from polyacrylamide gels to nitrocellulose sheets: Procedure and some application. *Proc. Natl Acad. Sci. USA*, **76,** 4350.